# Correlations between cortical gyrification and schizophrenia symptoms with and without comorbid hostility symptoms

**DOI:** 10.3389/fpsyt.2022.1092784

**Published:** 2023-01-04

**Authors:** Stefano Ferracuti, Antonio Del Casale, Andrea Romano, Ida Gualtieri, Martina Lucignani, Antonio Napolitano, Martina Nicole Modesti, Andrea Buscajoni, Teodolinda Zoppi, Georgios D. Kotzalidis, Lorenza Manelfi, Eleonora de Pisa, Paolo Girardi, Gabriele Mandarelli, Giovanna Parmigiani, Maria Camilla Rossi-Espagnet, Maurizio Pompili, Alessandro Bozzao

**Affiliations:** ^1^Department of Human Neuroscience, Faculty of Medicine and Dentistry, Sapienza University, Rome, Italy; ^2^Unit of Risk Management, Sant’Andrea University Hospital, Rome, Italy; ^3^Department of Dynamic and Clinical Psychology, and Health Studies, Faculty of Medicine and Psychology, Sapienza University, Rome, Italy; ^4^Unit of Psychiatry, Sant’Andrea University Hospital, Rome, Italy; ^5^Department of Neuroscience, Mental Health and Sensory Organs, Faculty of Medicine and Psychology, Sapienza University, Rome, Italy; ^6^Unit of Neuroradiology, Sant’Andrea University Hospital, Rome, Italy; ^7^IRCCS Bambino Gesù, Rome, Italy; ^8^Department of Interdisciplinary Medicine, Section of Criminology and Forensic Psychiatry, University of Bari, Bari, Italy

**Keywords:** schizophrenia, psychosis, neuroimaging, gyrification, magnetic resonance imaging, hostility

## Abstract

**Introduction:**

Interest in identifying the clinical implications of the neuropathophysiological background of schizophrenia is rising, including changes in cortical gyrification that may be due to neurodevelopmental abnormalities. Inpatients with schizophrenia can show abnormal gyrification of cortical regions correlated with the symptom severity.

**Methods:**

Our study included 36 patients that suffered an acute episode of schizophrenia and have undergone structural magnetic resonance imaging (MRI) to calculate the local gyrification index (LGI).

**Results:**

In the whole sample, the severity of symptoms significantly correlated with higher LGI in different cortical areas, including bilateral frontal, cingulate, parietal, temporal cortices, and right occipital cortex. Among these areas, patients with low hostility symptoms (LHS) compared to patients with high hostility symptoms (HHS) showed significantly lower LGI related to the severity of symptoms in bilateral frontal and temporal lobes.

**Discussion:**

The severity of psychopathology correlated with higher LGI in large portions of the cerebral cortex, possibly expressing abnormal neural development in schizophrenia. These findings could pave the way for further studies and future tailored diagnostic and therapeutic strategies.

## Introduction

Schizophrenia is a heterogeneous mental disorder characterized by exacerbating and remitting positive symptoms, coexisting with the so-called negative symptoms, which are relatively unremitting and resistant to drug treatment, and general symptoms and cognitive deficits, usually chronic (1, 2). Although challenging for clinical and research settings, investigating hostility and aggression in schizophrenia is difficult due to multi-factorial correlations and inconsistencies in the definition and assessment of these dimensions (3). Impaired disease awareness and impaired perception of the need for treatment are standard features of these patients’ clinical presentation.

Hostility implies the recurrent and persistent tendency to feel anger toward and a wish to injure a person or group. It can be related to a negative cognitive bias of strong dissatisfaction toward others with thoughts and feelings of antagonism, resentment, and alienation (3, 4) with a predisposition to attack one or more people in a connection. Patients with schizophrenia can show hostility symptoms both during the acute episodes and in stable phases of the disorder (3, 5). In some cases, hostility can lead to verbal or physical aggressiveness, and extensive population studies have shown a significant association between schizophrenia and violent behaviors (6–8). Furthermore, in patients with stable schizophrenia, excitement symptoms and prefrontal dysfunction correlated with elevated aggressiveness (9). These aspects are generally associated with poor compliance with treatment and involuntary hospitalization (10), which occurs more frequently in patients with schizophrenia than in patients with other mental disorders (11). Hostility-related dimensions in schizophrenia were related to structural and functional changes in different brain areas, including the prefrontal, anterior cingulate, and insular cortices, and amygdala, striatum, and hippocampus. These changes were involved in modulating hostile thoughts and behaviors, mainly in patients affected by schizophrenia with high urgency, impulsivity, and aggressiveness (3).

In recent decades, an increasing interest in identifying different aspects of mental and neurodevelopmental disorders through reliable magnetic resonance imaging (MRI) correlates has been observed, including the possible definition of their clinical implications (12). Gyrification is the result of the fetal and infantile developmental processes of gyri and sulci folding that transform the anatomy of the cortex from a smooth (“lissencephalic”) structure to a highly convoluted surface (13–15). The gyrification index (GI) is the ratio between the total contour of the pial surface and the contour of the smooth external surface (16–18) and increases with the complexity of the folds. It was initially calculated on two-dimensional coronal sections (16), but newer three-dimensional computations were developed and allowed for greater precision (19), representing an improvement over older methods (20).

The Local GI (LGI) is a measure based on voxel-based morphometry (VBM) techniques. LGI measures are currently widely accepted and involved in psychiatric studies, especially early psychotic episodes, depression, intellectual disabilities, 22q11 deletion, and schizophrenia (21, 22). Different studies demonstrated increased gyrification patterns (hypergyria) in individuals with autism (23–26), reduced gyrification in psychosis risk syndrome (27), and variable changes in gyrification patterns during the course of schizophrenia (16, 28, 29), which lead to the hypothesis that this is a morphological trace of early developmental anomalies that eventually persist throughout adulthood.

Though research has dedicated to the study of gyrification in the context of first-episode schizophrenia, chronic schizophrenia, siblings and at-risk individuals, findings are conflicting, and still need to clarify whether LGI can correlate with different aspects of the disease as well. To summarize the results of the LGI studies, it is thought that a characteristic of cortical gyrification in schizophrenia is a decreased GI and hypogyria. Abnormal cortical gyrification is also found in siblings of patients with schizophrenia and “high-risk” (HR) individuals who have family members with schizophrenia (21).

Gyrification is a potent marker of early neurodevelopment. It can be hypothesized that most of the gyrification process occurs during the third trimester of pregnancy, a period of considerable brain growth (30), and its pattern remains stable after birth (18, 31). Therefore, gyrification is a significant indicator of neurodevelopment, which proceeds according to an ordered topographic model (32–34).

Considering the neurodevelopmental hypothesis of schizophrenia (2, 35–37), early hypergyria would fit well with the abnormal mechanical developmental patterns of the brain. Furthermore, aberrant cortical curvature could be associated with reduced cortical thickness due to centripetal forces generated by cortico-cortical connections in cerebral maturation (13). Since the brain is believed to operate through intricate networks of neuronal connections, it has been hypothesized that the destruction of connectivity within a network or between two or more neural networks can be a major biological correlate of schizophrenia (38–41).

Differences in gray matter neuroanatomy and white matter connectivity are closely related. They may have common etiological pathways (18, 42): therefore, alterations of gyrification pattern could play a role in cognitive and psychiatric outcomes (43) and schizophrenia onset (29). There is a direct relationship between neural migration disorders (such as lissencephaly) and aberrant neural connectivity (44).

Hostility in schizophrenia is a critical clinical aspect, and there is a need for further neuroimaging studies on these dimensions. In this sense, hostility and/or aggressive behavior can manifest both as a psychopathological marker (lack of insight, medication non-adherence) and/or as a consequence of the disease (due to substance abuse and/or violence abuse) (45). Research still needs to clarify whether LGI can be a marker of hostility as a comorbid behavioral change in schizophrenia.

This study hypothesizes that patients affected by schizophrenia might show abnormal gyrification of specific cortical brain areas correlated with the symptom severity. Furthermore, LGI might show differences in patients with schizophrenia and high hostility symptoms (HHS) (in terms of BPRS hostility scores and history of compulsory treatment) when compared to patients with low hostility symptoms (LHS). We compared subjects dividing them into the two groups assessing them on clinical and neuroradiological bases, i.e., the LGI.

## Materials and methods

This is an observational, retrospective, non-interventional, single-center study focusing on the neuropathological correlates of LGI in patients with schizophrenia spectrum disorders, dividing them into HHS and LHS. The local ethical committee approved this study (prot. N. 470/2012). All subjects gave their informed consent before their inclusion in the study, which followed the generally accepted ethical research standards of the Declaration of Helsinki (46).

Patients admitted to the Psychiatric Diagnosis and Treatment Service of the “Sant’Andrea” University Hospital of Rome were included with the following criteria: diagnosis of non-affective psychosis at onset or schizophrenia, age range 18–65 years, and voluntary adherence to the research protocol. The treating psychiatrists established diagnoses according to the DSM-IV-TR criteria (47) and DSM-5 (48), which we confirmed according to DSM-5 criteria through medical record examination. Exclusion criteria were as follows: inability to undergo MRI examination, any neurological disease involving the central nervous system, history of traumatic brain injuries, radiological signs of neurological and neurovascular disease, untreated systemic illnesses, substance use disorder, and pregnancy.

### Instruments

We assessed psychiatric symptoms severity with the 24-item Brief Psychiatric Rating Scale, Expanded (BPRS-E) of Ventura et al. (49) in its validated Italian version (50) and related five-factor solution (51). The BPRS-E consists of 24 items, each to be rated by the clinician in a 7-point likert scale, ranging from “not present” to “extremely severe.” The five-factor solution we used was specifically investigated in a sample of involuntarily hospitalized inpatients, which assesses “mania/excitement,” “depression/suicidality,” “hostility,” “positive symptoms,” and “negative symptoms.” This solution showed good reliability and validity (49).

For each hospitalization, we evaluated the following clinical and socio-demographic variables: age, gender, disease duration, and days of hospitalization. In addition, we considered the type and dosage of antipsychotics on admission. To allow comparisons, the dosage of all antipsychotics was converted to chlorpromazine equivalents. Concomitant intake of other drug classes (benzodiazepines, antidepressants, mood stabilizers) was also annotated. 7 patients were on concomitant antiepileptic treatment, while 6 were on concomitant benzodiazepines treatment. No patients were treated with Long-Acting Injection (LAI) antipsychotics. We then divided all patients into two groups, according to the BPRS scores, in HHS, including patients who had a BPRS hostility factor score major than the mean score of the whole sample (15.8) and a history of compulsory treatment, and LHS, including patients with lower BPRS hostility scores without a history of mandatory treatment.

### MR imaging acquisition

All participants underwent structural MRI (1.5 T; MP-RAGE 3D sequences) T1-weighted 3D magnetization-prepared rapid gradient echo (MP-RAGE) sequences (TR = 1,100 ms, TE = 3.49 ms, TI = 600 ms, FA = 15°, ST = 1 mm) were collected on a 1.5T scanner (Magnetom Sonata, Siemens, Erlangen, Germany).

### Image analysis

Data were processed with FreeSurfer 7 software^1^ (52), using a standard automatic pipeline (i.e., recon-all) that sequentially performed skull stripping, intensity correction, and transformation to Talairach-Tournoux space to produce grey matter (GM) and white matter (WM) segmentation. The GM–WM boundary was first determined and then tessellated to generate the inner cortical surface (white surface) by combining tissue intensity and neighborhood constraints information. The outer surface (pial surface) was then generated through the expansion of the white surface with a point-to-point correspondence. Finally, according to the approach described by Fischl and Dale (53), LGIs were computed vertex-wise over the entire cortex using the method of Schaer et al. (54), which measures the amount of cortex buried within the sulcal folds as compared with the amount of visible cortex in spherical regions of interest. For statistical purposes and visualization, we resampled cortical parameters onto a common surface template provided by FreeSurfer.

### Statistical analysis

Inter-group differences were analyzed through the analysis of variance. To this end, we mapped vertex-wise LGI values on a common spherical coordinate system (i.e., fsaverage) using spherical transformation. We assessed differences among the groups in separate analyses, investigating HHS > LHS and HHS < LHS contrasts with permutation tests (1000 permutations for each test) based on t statistics. The Permutation Analysis of Linear Models (PALM) FSL package was employed [FMRIB Software Library v6.0 (55)]. Particularly, we used group illness duration (expressed in months) as a covariate to produce Threshold-Free Cluster Enhancement (TFCE) statistical maps, where the initial raw statistical images were enhanced using both the intensity of the data point and information from neighboring voxels (56). We detected group differences (HHS vs. LHS) on family-wise error (FWE) corrected p-value and uncorrected p-values maps. Correlation analyses were evaluated vertex-wise between LGI cortical parameter and the BPRS-E total score as clinical variable, testing Pearson correlation with PALM permutation test (1,000 permutations). Moreover, correlation analysis was also performed for those brain areas LGI values that resulted significantly different between HHS and LHS. Both correlation analyses were performed by thresholding for false-positive results with a FWE rate correction. Statistical results were displayed on a common inflated surface template.

## Results

### Clinical characteristics

Our study included 36 participants (10 women, 26 men; mean age = 26.53 years, SD = 7.43) affected by schizophrenia. We summarized the socio-demographic and clinical characteristics of the sample in Table 1.

**TABLE 1:** Sociodemographic and clinical characteristics of clinical samples.

	HHS	LHS	*F (χ^2^)*	*p*
Mean age (SD)	25.68 (6.18)	27.20 (8.39)	0.361	0.552
Gender (Male/female)	13/3	13/7	1.17	0.279
Mean illness duration, months (SD)	41.99 (52.16)	58.54 (83.25)	0.48	0.493
Mean duration of last hospitalization, days (SD)	18.76 (11.49)	12.23 (6.19)	4.004	0.055
Mean BPRS mania/excitement (SD)	25.68 (5.08)	20.85 (6)	6.597	**0.015**
Mean BPRS depression/suicidality (SD)	17.06 (3.91)	15.95 (4.08)	0.685	0.414
Mean BPRS hostility (SD)	19.31 (1.25)	13.1 (3.32)	50.01	**<0.001**
Mean BPRS negative symptoms (SD)	15.25 (2.14)	13.8 (4.03)	1.68	0.204
Mean BPRS positive symptoms (SD)	21.56 (2.76)	20.8 (2.8)	0.668	0.42
Mean BPRS total score (SD)	116.06 (7.27)	100.25 (7.76)	38.938	**<0.001**
Mean CPZ equivalents	68.20 (129.22)	245.38 (312.34)	4.208	**0.049**

HHS, high aggressive symptoms patients; LHS, low aggressive symptoms patients; BPRS, Brief Psychiatric Rating Scale; CPZ, chlorpromazine; SD, standard deviation. Bold indicates significant results for *p* < 0.05.

The BPRS-E scale in our study sample showed a good internal consistency (Cronback’s α = 0.572), as well as the related hostility factor (Cronback’s α = 0.806) that we used to define the two subsamples. LHS vs. HHS group showed significantly higher dosage of antipsychotics (chlorpromazine equivalents) (F = 4.208; *p* < 0.05), lower scores on the BPRS-E mania/excitement (F = 6.597; *p* = 0.015) and hostility (F = 50.01; *p* < 0.001) factors.

### Local gyrification results

#### Whole sample total BPRS-E/LGI correlation

**FIGURE 1:**
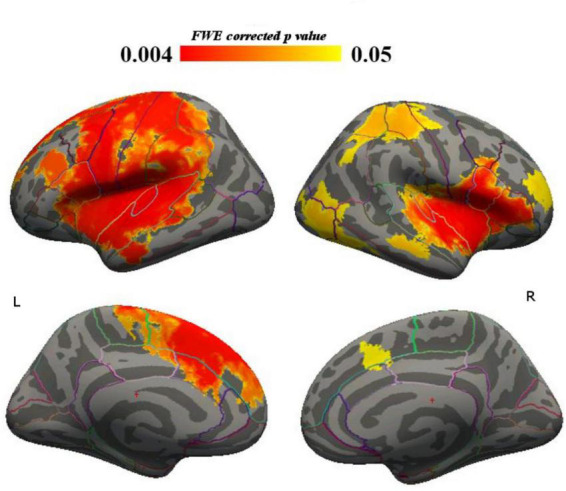
Total BPRS—LGI correlation, whole sample.

The BPRS-E total score significantly correlated with higher LGI in different cortical areas, including bilateral frontal, caudal anterior and posterior cingulate, parietal, temporal cortices, and right occipital cortex. We summarized these results in Table 2, reporting the main involved cortical areas for LGI-PBRS correlation, sorted for decreasing extent of significantly correlated vertices. Significant areas are displayed in Figure 1.

**TABLE 2:** LGI-BPRS correlation—whole sample.

	LGI-BPRS mean correlation (SD)	*P*-value	Main involved structures (number of significant vertices)
**Left brain**			
Frontal lobe	0.35 (0.04)	0.020	Pre central (10,455), superior frontal (10,397), post central (8,508), pars opercularis (3,085), caudal middle frontal (2,630), rostral middle frontal (2,123), paracentral (1,281), pars triangularis (781), lateral orbito frontal (408)
Limbic lobe	0.33 (0.03)	0.024	Caudal anterior cingulate (1,116), banks-sts (1,101), posterior cingulate (470)
Parietal lobe	0.33 (0.03)	0.023	Supramarginal (8,351), superior parietal (2,251), inferior parietal (454)
Temporal lobe	0.41 (0.04)	0.015	Superior temporal (6,505), insula (4,634), middle temporal (1,188), transverse temporal (1,064)
**Right brain**			
Frontal lobe	0.40 (0.05)	0.033	Post central (4,835), pre central (3,238), rostral middle frontal (2,811), pars opercularis (2,376), pars triangularis (2,222), superior frontal (1,540), caudal middle frontal (338), lateral orbito frontal (291), pars orbitalis (229), paracentral (186)
Limbic lobe	0.36 (0.02)	0.048	Caudal anterior cingulate (179), posterior cingulate (131)
Occipital lobe	0.40 (0.05)	0.043	Lateral occipital (2,047)
Parietal lobe	0.35 (0.03)	0.043	Superior parietal (2,872), inferior parietal (2,444), supramarginal (1,663)
Temporal lobe	0.40 (0.05)	0.033	Insula (4,614), superior temporal (4,201), middle temporal (1,078), inferior temporal (816), transverse temporal (781), fusiform (653)

BPRS, Brief Psychiatric Rating Scale; LGI, local gyrification index; SD, standard deviation.

#### Between-group differences in the BPRS-E/LGI correlation

**FIGURE 2:**
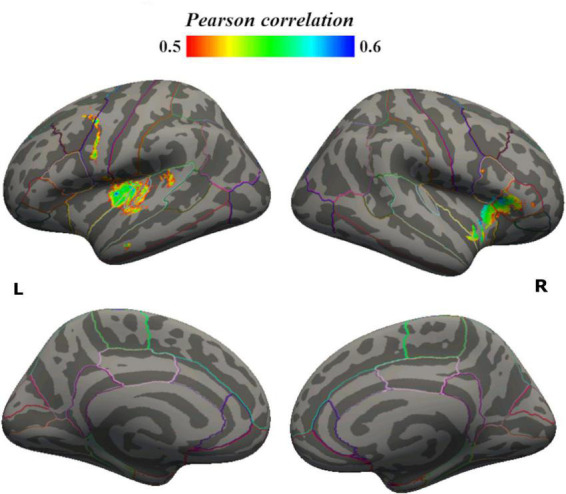
Total BPRS—LGI correlation, between-group differences.

Focusing on brain areas where LGI was significantly correlated with BPRS-E, we found reduced significant clusters within the bilateral frontal and temporal lobes in LHS when compared to HHS (Table 3 and Figure 2).

**TABLE 3:** LGI-BPRS correlation, between-group differences.

	LGI-BPRS mean correlation (p val.)	Mean LGI HHS	Mean LGI LHS (p val.)	Main involved structures (number of significant vertices)
**Left brain**				
Frontal lobe	0.51 (0.004)	4.11	3.90 (0.031)	Pre-central, post-central, caudal middle frontal
Temporal lobe	0.52 (0.05)	4.39	4.11 (0.038)	Insula, superior temporal, transverse temporal, middle temporal
**Right brain**				
Frontal lobe	0.55 (0.01)	4.59	4.24 (0.046)	Pars triangularis, pars opercularis, lateral orbito frontal
Temporal lobe	0.54 (0.01)	4.79	4.44 (0.045)	Insula, superior temporal

HHS, high aggressive symptoms patients; LHS, low aggressive symptoms patients; BPRS, Brief Psychiatric Rating Scale; LGI, local gyrification index.

#### Between-group comparison

**FIGURE 3:**
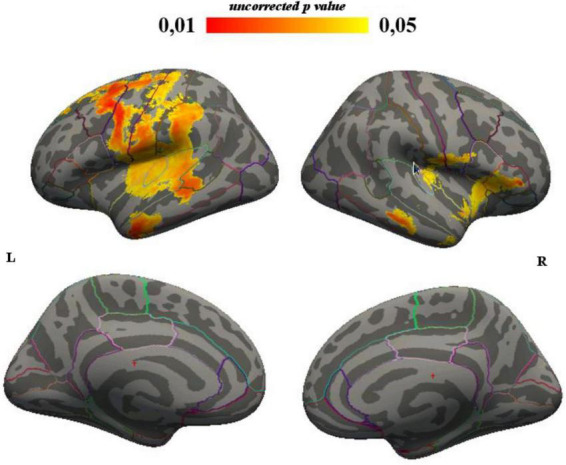
LGI, between-group differences corrected for illness duration (uncorrected p results).

Mean LGI distribution was computed for both hemispheres in HHS and LHS. Uncorrected p statistical analysis showed decreased LGI in LHS vs. HHS in bilateral frontal and temporal lobes and left parietal lobe (Table 4 and Figure 3). FWE correction showed decreased LGI in LHS vs. HHS of left-sided superior and transverse temporal cortices, and left insula (Table 5).

**TABLE 4:** HHS/LHS direct comparison for mean LGI, between-group differences (uncorrected *p*).

	HHS mean LGI (SD)	LHS mean LGI (SD)	*P*-value	Main involved structures (number of significant vertices)
**Left brain**				
Frontal lobe	3.35 (0.30)	3.22 (0.27)	0.040	Post central (5,598), pre central (5,340), caudal middle frontal (1,750), superior frontal (1,148)
Parietal lobe	3.52 (0.16)	3.40 (0.14)	0.043	Supramarginal (4,765), superior parietal (53)
Temporal lobe	4.00 (0.17)	3.80 (0.15)	0.037	Superior temporal (3,927), insula (1,904), middle temporal (1,324), transverse temporal (1,064), inferior temporal (284)
**Right brain**				
Frontal lobe	4.69 (0.15)	4.37 (0.12)	0.045	Pars triangularis (1,051), post central (457), pre central (450), pars opercularis (108)
Temporal lobe	4.56 (0.29)	4.29 (0.26)	0.046	Insula (952), middle temporal (585), superior temporal (119), transverse temporal (86)

HHS, high aggressive symptoms patients; LHS, low aggressive symptoms patients; LGI, local gyrification index; SD, standard deviation.

**TABLE 5:** HHS/LHS direct comparison for mean LGI - between-group differences (FWE p).

	# vertices involved	Mean *P*-value	HHS mean LGI (SD)	LHS mean LGI (SD)
LH superior temporal	539	0.082	4.143 (0.086)	3.902 (0.078)
LH transverse temporal	83	0.082	4.538 (0.048)	4.269 (0.036)
LH insula	222	0.082	4.613 (0.058)	4.345 (0.059)

FWE, family-wise error; LGI, local gyrification index; HHS, high aggressive symptoms patients; LHS, low aggressive symptoms patients; LH, left hemisphere; SD, standard deviation.

## Discussion

Our correlation analyses of the whole sample showed that the severity of symptoms significantly directly correlated with the LGI in different cortical areas, including bilateral frontal, cingulate, parietal, temporal cortices, and right occipital cortex. This is partly in line with a recent study that showed a symptom-LGI direct correlation in patients with a first episode of schizophrenia in the left temporal cortex (57). The involvement of more cortical brain areas in our study could be due to the differences in the sample compositions. The picture of a diffuse hypergyria associated with schizophrenia, regardless of the correlation with the severity of symptoms, is reported by several studies, and our results are to be considered in the context of this evidence, mainly during the illness onset (58–60).

Considering the lack of MRI correlation studies on more advanced stages of schizophrenia, an issue that needs more investigation relates to whether the correlation between LGI and symptoms severity is a constant aspect of the disease or whether it is more typical of the onset and early course of schizophrenia (29). Further longitudinal, case-control, and symptom correlation neuroimaging studies are needed. These may shed light on the nature and clinical correlates of gyrification changes in schizophrenia, also considering that future studies could take advantage of higher magnetic induction fields (≥3 T), which could reinforce existing evidence or lead to more accurate findings.

We found that LHS showed significantly lower LGI related to the severity of symptoms in the bilateral frontal lobe, including the right pars triangularis, opercularis, and orbitofrontal cortex, and left precentral, postcentral, and caudal middle frontal cortices. Furthermore, we observed the same reduction in LHS in bilateral temporal areas, including the bilateral insula, bilateral superior temporal cortices, left transverse and middle temporal cortices.

The insular cortex plays an essential role in the consciousness processes of one’s body and its properties (61–64), emotions (65), empathy (66), sense of agency (67), and language (68, 69). The insula is thought to be involved in the violation of rules and social conventions as well (70), together with processing convergent information to produce a relevant emotional context in response to sensory experience and in a general salience system that monitors the surrounding environment and selects appropriate responses and behaviors (71).

Other studies have reported an association between destructive, aggressive, and antisocial behaviors and increased gyrification of the insula and superior temporal gyrus (72, 73). These clinical factors have been associated with involuntary psychiatric hospitalization (74–76). Dysfunction of neural connectivity in the left insula has been hypothesized to represent neural correlates of clinical insight impairment in schizophrenia spectrum disorders (77). Our results can be linked with existing evidence of insular structural and functional changes mirroring poor illness insight in patients with schizophrenia (78, 79).

Temporal regions also play an essential role in different dimensions of insight (80), and insight deficits have been previously associated with structural alterations of the temporal cortex (81, 82). Another important study showed that gyrification patterns in schizophrenia spectrum disorders in frontal and temporal areas are related both to early neurodevelopmental abnormalities (vulnerability) and active brain pathology, especially in the early stages of illness (83).

We showed gyrification changes in the right orbitofrontal cortex in patients affected by schizophrenia with hostility/aggressive symptoms. This area has been involved in the development of severe negative symptoms (84), deficits in abstract thinking (85), and could be interpreted in the context of the evidence of a structural deficit in the corticothalamic systems, especially in the orbitofrontal-thalamic system in schizophrenia (86). We also discovered a lower LGI/symptom correlation of the right pars triangularis in the LHS vs. HHS group, therefore confirming the existing evidence of structural and functional deficits in this area in patients with schizophrenia and states of chronic illness with a lack of medication (87), cognitive deterioration (88), lack of cognitive insight (89), and symptom misattribution (90). Furthermore, a significantly impaired brain activity of the right pars triangularis in response to auditory words presented with negative valence has been crucially involved in the neuropathophysiology of positive symptoms, including the persecutory delusion and delusional behavior (91). These findings are in line with our results, suggesting an involvement of the right pars triangularis in the neuropathophysiology of severe symptoms of schizophrenia.

HHS patients showed significantly lower dosages of antipsychotics on admission than their counterparts. This result might also be interpreted in the light of poor adherence to treatments before hospitalization, i.e., one of the most important factors that usually lead to compulsory hospitalization (87). This is in line with other studies highlighting the risk of aggression toward others (74, 75) and psychomotor agitation (76) as factors strongly associated with involuntary hospitalization.

This study showed a correlation between the severity of psychopathology and cortical gyrification in schizophrenia, both in LHS and HHS. One explanation for this finding is that a higher level of gyrification correlates with neurodevelopmental impairment, which predisposes patients to manifest more severe psychotic episodes during schizophrenia. Poor insight and aspects that may arise from it, including involuntary hospitalization, treatment non-adherence, lower psychosocial functioning, poor prognosis, and higher utilization of emergency services, are all clinical issues hypothesized to have neurobiological bases (92). Current evidence identified specific neural correlates for insight types and dimensions, such as anatomical and functional changes in the prefrontal cortex, cingulate cortex, and regions of the temporal and parietal lobe (precuneus, inferior parietal lobule), and hippocampus (80). Another point of reflection for future research is provided by the possible association between gyrification and aggressiveness in schizophrenia. An element that could be analyzed would be gender differences in gyrification, considering that male patients with schizophrenia could manifest aggressiveness more often than female counterparts (93). Greater severity of psychopathology, also related to LGI changes, can predispose patients to manifest symptoms that are risk factors for compulsory treatment, for example, acute worsening, aggressive behaviors, social maladjustment, poor therapeutic compliance, and prolonged hospitalizations (94). The results of this study, even if of an exclusively biological nature, may pave the way for further studies and the acquisition of big data, which may in the future have a clinical implication using clinical applications, which may help to guide the diagnostic and treatment pathways in patients with acute schizophrenia (95). Other important clinical perspectives regard the possibility to overcome to LGI impairment through the restoration of brain activity via brain stimulation. Initial evidence is now available regarding the possibility to modulate aggression/impulsivity features in several psychiatric disorder thanks to Brain stimulation techniques (96–98).

### Limitations

The major limitations of this study consist in its retrospective and transversal nature and small sample. Another limitation could be seen in the lack of a gold standard measure of clinical severity (i.e., Clinical Global Impression). However, the sample consists of inpatients with acute schizophrenia, most with high scores on the CGI scale, which would have resulted in a flattening of the variance with reduced possibilities of observation. The last limitation concerns not having performed controlled statistics with respect to antiepileptic drugs treatment (7 subjects). Nevertheless, most of them had been prescribed antiepileptics at the beginning of the last hospitalization, therefore it is very unlikely that cerebral morphology could have been affected by these medications in very few days. Results should be taken with caution and need to be replicated, also based on longitudinal studies that could shed light on the nature and clinical correlates of these biological aspects.

## Conclusion

Acute inpatients with schizophrenia showed increased LGI correlated to symptom severity in bilateral frontal, cingulate, parietal, temporal cortices, and right occipital cortex. The comparison between LHS and HHS showed in the first group a significantly lower LGI related to the severity of symptoms in the bilateral frontal and temporal cortices.

These changes in LGI involve abnormalities in areas that have been already associated with a lack of insight and awareness of the illness, severity of psychotic symptoms, and aggressive and antisocial behavior, which are frequently related to involuntary hospitalization. Consequently, the LGI differences found between HHS and LHS could be mediated by the severity of psychopathology. Gyrification changes can be a measurable correlate, possibly linking neurodevelopment alterations to specific clinical dimensions in adults who have schizophrenia. The modifications in LGI we found in HHS might represent a new possible biological component for severe clinical behavioral disturbance. Further longitudinal studies are needed to better understand the nature of these changes and their possible correlation in the context of severe schizophrenia.

## Data availability statement

The raw data supporting the conclusions of this article will be made available by the authors, without undue reservation.

## Ethics statement

The studies involving human participants were reviewed and approved by the Sapienza University Ethics Committee prot. N. 470/2012. The patients provided their written informed consent to participate in this study.

## Author contributions

SF and ADC ideated and designed the study. ADC, AnB, EP, GM, GP, IG, LM, PG, and TZ performed the clinical data analyses. AR and MR-E performed the MRI acquisition and data collection. ML, AN, ADC, and MR-E conducted the statistical analyses. SF, ADC, IG, MNM, and GDK provided the first draft. ADC, GDK, SF, MP, and AlB supervised the writing of the manuscript. All authors contributed to the manuscript writing and approved the final draft.

## Footnotes

1
https://surfer.nmr.mgh.harvard.edu/
